# Daily Stress Variability in Two Generations of Survivors of the War in the Former Yugoslavia

**DOI:** 10.1002/smi.70113

**Published:** 2025-10-16

**Authors:** Nikola Doubková, Filip Zlámal, Monika Fňašková, Marek Preiss, Markéta Nečasová, Nikola Wolframová, Vojtěch Svoboda, David Ulčák, Ivan Rektor

**Affiliations:** ^1^ National Institute of Mental Health Klecany Czech Republic; ^2^ Central European Institute of Technology (CEITEC) Centre for Neuroscience Masaryk University Brno Czech Republic; ^3^ Faculty of Sports Studies Department of Physical Activities and Health Sciences Masaryk University Brno Czech Republic; ^4^ University of New York in Prague Prague Czech Republic; ^5^ Institute of Physiology Medical University of Innsbruck Innsbruck Austria; ^6^ St. Anne's University Hospital and Faculty of Medicine Department of Neurology Masaryk University Brno Czech Republic

**Keywords:** experience sampling method, latent vulnerability hypothesis, stress, stress variability, trauma, war survivors, Yugoslavia War

## Abstract

The war in the former Yugoslavia had a profound impact on millions of civilians, leaving long‐lasting psychological consequences. This study aimed to examine stress sensitivity and variability in the daily lives of survivors using a longitudinal design. First‐generation survivors (G1; *n* = 79), second‐generation survivors born after the war (G2; *n* = 28), and a non‐war‐exposed control group (*n* = 60) participated. The baseline assessment included measures of stress‐ and trauma‐related symptoms, life satisfaction, and coping mechanisms. Daily perceived stress was then monitored over 21 consecutive days using the experience sampling method. Although there were no group differences in baseline measures or mean daily stress levels, variability in daily stress showed distinct generational patterns. G1 exhibited lower variability compared to both controls and G2, which showed the highest variability. Variability was significantly associated with trauma‐related symptoms, dysfunctional coping, and life satisfaction. This study showed that the lasting psychological consequences of the war in the former Yugoslavia may not be reflected in elevated daily stress levels or baseline psychopathology but rather may be subtly expressed through altered perceptions and sensitivity to daily stress, even decades after the war. These findings provide novel support for the latent vulnerability hypothesis.

## 1 Introduction

Undoubtedly, war has lasting psychological consequences on civilians (Priebe et al. 2013). Increased prevalence of posttraumatic stress disorder (PTSD), mood, and anxiety disorders was reported in people exposed to armed conflicts (Carpiniello 2023; Jong et al. 2003; Murthy and Lakshminarayana 2006). The consequences of war experiences are truly long‐lasting, as they may affect the mental health of survivors even 50 years after the war and subsequent generations as well (Betancourt 2015; Bramsen and Ploeg 2007; Shrira et al., 2024b).

One of the most devastating armed conflicts in Europe since the end of World War II was the war in the former Yugoslavia, which began in 1991 and officially ended in 1995 with the Dayton Agreement. However, the reverberations of the war persisted until the early 2000s, with significant events such as the war in Kosovo, during which the North Atlantic Treaty Organisation (NATO) conducted aerial bombardment of the former Yugoslavia in 1999. During the war in the former Yugoslavia, tens of thousands of people were killed as a result of combat and ethnic cleansing (e.g., Srebrenica) (Sorgo and Zivkovic 2015).

Due to the war in the former Yugoslavia, civilians were exposed to several potentially traumatic events and often endured multiple such events during that period (e.g., Comtesse et al. 2019; Nickerson et al. 2014; Priebe et al. 2013). The frequency of potentially traumatic experiences varied slightly among the affected territories (Priebe et al. 2010); however, the most common experiences were shelling or bombardment, affecting approximately 85% of people, followed by a lack of shelter, which impacted around 51% of people, and approximately 38% of civilians were expelled from their homes under threat (Nickerson et al. 2014; Priebe et al. 2010, 2013).

Almost two million people were displaced, with more than half emigrating abroad (Sorgo and Zivkovic 2015). This resulted in a cumulative burden of war‐related, migratory, and post‐migratory trauma that can have severe psychological consequences on affected civilian survivors, such as PTSD years later (Bogic et al. 2012; Carpiniello 2023; Priebe et al. 2009). Facing migration‐related stressors such as legal insecurity, lack of economic opportunities, and not feeling accepted represents additional burdens that might further exacerbate psychopathology symptoms (Bogic et al. 2012; Matanov et al. 2013). Consequently, significantly higher health risks were found in migrants from the former Yugoslavia compared to other migrants from the same period in Sweden, including an increased likelihood of being diagnosed with somatic diseases such as cardiovascular or cancer, but also mental disorders and particularly PTSD (Thordardottir et al. 2020). Another study (Priebe et al. 2009) found that 83.7% of 264 war survivors living in Croatia, Serbia, Germany, and the United Kingdom who never received treatment fulfiled diagnostic criteria for PTSD. Both studies assessed survivors of the war in the former Yugoslavia approximately a decade after the war yet still found high rates of PTSD and other mental health difficulties. Studies also found that these enduring consequences of surviving war extend beyond the initial event and shape cognitive responses, interpersonal interactions and sensitivity, overall quality of life and satisfaction, and mental health functioning, further influencing how survivors navigate their daily lives (Matanov et al. 2013; Munjiza et al. 2017; Nickerson et al. 2014).

Even though the psychological scars of war are undeniably severe, most people who experience trauma show the capacity to grow and the ability to restore well‐being and functioning despite adversity (Hamby et al. 2018; Kelmendi and Hamby 2023; Saar‐Ashkenazy et al. 2024). Numerous psychosocial factors contribute to resilience after trauma exposure (Hamby et al. 2018; Iacoviello and Charney 2014; Kelmendi and Hamby 2023; Saar‐Ashkenazy et al. 2024). Among these, coping skills, incorporating cognitive, affective, and behavioural components, are crucial (Carver et al. 1989; Lazarus and Folkman 1984). Active coping mechanisms, such as planning, seeking instrumental and emotional support, religion, and humour, are associated with resilient outcomes. In contrast, dysfunctional coping mechanisms, such as denial, self‐blame, or substance use, increase the likelihood of psychopathology (Iacoviello and Charney 2014).

However, the adaptiveness of coping strategies is highly context‐dependent; strategies often considered dysfunctional may be adaptive under certain conditions, and vice versa (Ben‐Zur and Zeidner 1995; Carver et al. 1989; Saar‐Ashkenazy et al. 2024). A scoping review (Kelmendi and Hamby 2023) illustrates these findings in the cultural context of Kosovo and Southeast Europe. The review identified key coping mechanisms positively associated with resilience and lower distress levels, including problem‐solving, cognitive flexibility, shifting from negative to positive thinking, and communal coping through collective responses. However, findings about emotional suppression are mixed. On the one hand, it was suggested as a culturally relevant positive coping mechanism connected to regulatory strengths, endurance, honour, and dignity. On the other hand, a negative correlation with positive outcomes was found as it was linked to avoidance, disengagement, and substance use (Anderson et al. 2019; Kelmendi and Hamby 2023).

According to the historical trauma theory, significant events such as the war in the former Yugoslavia affect not only those directly exposed but also entire communities and social structures. Moreover, the theory presumes that the emotional and psychological consequences can be transmitted to subsequent generations even if the direct war experience is absent (Danieli 1998; Hartmann and Gone 2014). Various possible psychological, behavioural, and physiological (e.g., epigenetic) transmission paths have been described (Bar‐On et al. 1998; Bowers and Yehuda 2016; Lehrner and Yehuda 2018; Preiss and Biran 2023; Sales and Fivush 2005; Sotero 2006). Importantly, transmission can involve not only vulnerabilities, stress, and trauma‐related symptoms but also strengths, resilience, and coping mechanisms (Bowers and Yehuda 2016; Fňašková et al. 2021; Harvey 2007; Maxwell 2014; Payne and Berle 2021; Scharf 2007; Van IJzendoorn et al. 2003). There is a lack of studies assessing intergenerational phenomena among the survivors of the war in the former Yugoslavia (Hyseni Duraku et al. 2023; Priebe et al. 2010). Studies have found heightened stress sensitivity and increased risk of PTSD symptoms in the second generation of survivors, which are likely influenced by intergenerational transmission, suppressed family narratives, and parental psychopathology (Dikyurt 2023; Hyseni Duraku et al. 2023; Roch 2023).

The phenomenology of how these consequences reverberate across generations is heterogeneous, and the studies of descendants are somewhat inconclusive regarding their mental health (Payne and Berle 2021; Shrira et al. 2025; Van IJzendoorn et al. 2003). Thus, the latent vulnerability hypothesis (LVH; Shrira 2015) has been proposed to understand how ancestral trauma impacts descendants. The LVH states that parental traumatic experiences lead to increased vulnerability to stress and psychopathology in their descendants. More concretely, it suggests that the mental health difficulties of descendants are latent in low‐stress conditions, and the vulnerability manifests in increased psychological distress when exposed to stressful or potentially traumatic events (Shrira 2015; Shrira et al. 2025).

It was found that descendants of Holocaust survivors in Israel exhibit heightened stress reactivity to stressful or traumatic events compared to control groups, particularly when faced with adversities that resemble their ancestral trauma (Shrira et al. 2025; Shrira, Greenblatt‐Kimron, et al., 2024). The LVH has received empirical support, primarily in studies of descendants of Holocaust survivors (Greenblatt‐Kimron et al. 2023; Shrira et al. 2024b, 2025; Shrira and Felsen 2021) and, to a much lesser extent, in other survivor groups, such as children of United States soldiers who served in the Vietnam War (Rosenheck and Fontana 1998) and Hiroshima and Nagasaki survivors (Palgi et al. 2012). Despite the evidence supporting the LVH, further longitudinal studies are needed to examine second‐generation individuals from other trauma‐affected groups as well as how vulnerability unfolds in daily life. To date, most studies have focused on responses to events rather extreme in nature, leaving everyday processes unexplored.

The present study focused on the long‐term psychological impact of war‐related stress on survivors of the war in the former Yugoslavia (first‐generation survivors, G1) and the second‐generation (G2) born after the war. Specifically, the study aimed to explore stress sensitivity and variability in daily life using a longitudinal design, that is, the experience sampling method (ESM; Bolger and Laurenceau 2013; Hektner et al. 2007; Larson and Csikszentmihalyi 2014). Based on the LVH, which posits that latent stress vulnerability manifests as heightened reactivity when stressors occur, we hypothesised that, while average daily stress levels might not differ between groups, G2, in particular, would show heightened sensitivity to stressors, reflected in greater variability in perceived daily stress. Additionally, given the potential intergenerational effects, we expected G1, G2, and the non‐war‐exposed control group to differ in baseline psychological factors—including the severity of stress and trauma‐related symptoms, coping styles, and life satisfaction—and that these would also play a role in shaping perceived daily stress. By integrating longitudinal methods with a baseline psychological assessment, the study aimed to contribute to the field by identifying vulnerability and resilience patterns in a previously understudied war‐affected population.

## 2 Methods

### 2.1 Participants and Procedure

This study was conducted according to the guidelines of the Declaration of Helsinki. Ethical approval for this study was obtained from the ethics committee of Masaryk University (approval code EKV‐2021‐076) on June 24, 2021. All participants and/or their legal guardians were informed about the study procedures and objectives before participating and signed an informed consent form.

A power analysis was conducted to determine the sample size needed for this study. Based on a previous Czech study comparing stress and trauma‐related symptoms in Holocaust survivors to a control group (Fňašková et al. 2021), at least 30 participants in each group are needed to detect group differences with a statistical power of 0.80 and a significance level of 0.05.

Data was collected between May 2022 and August 2024. All participants met face‐to‐face with research team members, were informed about the goals and procedures of the study and signed a written informed consent form. During a four‐to five‐hour session, participants underwent a physiological and neuroimaging assessment, followed by a semi‐structured interview, and subsequently completed questionnaires. Afterwards, participants were enroled in the 21‐day‐long ESM part of the study using their smartphones. Daily questionnaires were distributed using SoSci Survey (Leiner 2019) and SurveySignal (Hofmann and Patel 2015). All volunteers were reimbursed for transport costs.

In total, 179 people consented to participate; however, 12 were excluded from the analysis because they did not complete the ESM part of the study (e.g., declined after baseline, experienced technical difficulties). Thus, 167 participants were included, 55.1% of whom were women, and their ages ranged from 17 to 71 years. The participants were divided into three groups:

1. First generation (G1): Participants who directly experienced the war in the former Yugoslavia during the 1990s and emigrated to the Czech Republic either directly or via another country (*n* = 79, 49.4% women, M[SD]_age_ = 37.1[8.4], age range: 24–71 years). A majority were married or in a partnership (59.5%), and 60.8% reported an above‐average monthly income relative to the national median.
2. Second generation (G2): Biological children of survivors of the war in the former Yugoslavia, born after the conflict (post‐1995) and residing in the Czech Republic at the time of the study (*n* = 28, 67.9% women, M[SD]_age_ = 24.5[2.1], age range: 22–29 years). Most participants were single (78.6%), with their monthly income mostly rated as below average (39.3%) or average (28.6%); 28.6% chose not to report income. Due to ESM attrition, the sample size of this group was slightly below the recommended power analysis benchmark; however, this was partly offset by the repeated‐measures design.
3. Non‐war‐exposed control group: Included were participants without personal or immediate family history of war/combat exposure or displacement, recruited with comparable age and gender distribution to the survivor groups (*n* = 60, 56.6% women, M[SD]_age_ = 33.6[11.6], age range: 17–67 years). Most participants were single (65%). Income levels varied, with 51.7% reporting below average, 21.7% average income, 21.7% above‐average income, and 4.9% choosing not to disclose their income.

General exclusion criteria included brain impairments (e.g., brain injuries or diseases, tumours, neurodegenerative diseases), severe mental disorders (e.g., psychosis), and significant cognitive decline.

### 2.2 Measures

English or Czech version of the following questionnaires was used:

*Demographic information* was collected, including gender, age, relationship status, and self‐reported income relative to the national median, as well as war‐related background (e.g., personal and parental exposure to war, and migration history).

*PTSD Checklist for DSM‐5* (PCL‐5; Weathers et al. 2013) is a self‐report measure assessing the severity of symptoms of PTSD according to the DSM‐5. The participants are asked to rate how much they were bothered by each of the 20 symptoms using a five‐point Likert scale from 0 (not at all) to 4 (extremely). The total score is obtained by summing the score for each item and ranges between 0 and 80. Cronbach's α was good (*α* = 0.92).

*Satisfaction with Life Scale* (SWLS; Diener et al. 1985) is a five‐item self‐report scale that measures global life satisfaction. The total summing score ranges between 5 and 35. Cronbach's α was good (*α* = 0.84).

*Brief COPE* (Carver 1997) assesses three overarching coping styles with adversities: (1) Problem‐focused (e.g., planning, use of instrumental support), (2) Emotion‐focused (e.g., positive reframing, humour), and (3) Dysfunctional coping (e.g., denial, disengagement) (Coolidge et al. 2000). It includes 28 self‐report items. Scores are given by the average of items for the three coping styles and range between 1 and 4. Cronbach's α was acceptable to good (Problem‐focused: *α* = 0.77, Emotion‐focused: *α* = 0.66, Dysfunctional: *α* = 0.72, and *α* = 0.84 for the whole scale).

For the ESM part of the study, the *Perceived Stress Scale* (PSS; Cohen et al. 1983), which assesses the perception of stress and the degree to which recent experiences exceed the adaptive capacities of the participants, was adapted for daily use in the random time in the evening for 21 consecutive days. The original PSS asks respondents to evaluate how often they experience certain feelings or thoughts during the past month. In our study, the PSS instructions were reframed to: ‘Today, how much have you felt…’. Each of the 10 items was rated on a 5‐point Likert scale from 0 (very slightly or not at all) to 4 (extremely), that is, higher scores indicate higher perceived stress.

The modifications of the PSS were based on the study by Murray et al. (2023) that used a four‐item version of the PSS and collected data four times a day for 14 consecutive days. Given the differences in study designs and aims, we retained the original 10‐item version but modified the instructions and Likert‐scale labels to align with the approach of Murray et al. (2023).

In our study, Cronbach's α was good (*α* ranging between 0.85 on day one to 0.93 on day 14). The average time of responding was 55.5 s, and the average time needed for responding decreased over time (from 78.6 s on day 1 to 46.6 s on day 20; however, after day 11, the time needed for responding became more stable, i.e., between 51.7 and 46.5 s). The average response rate (number of completed questionnaires/days) was 69%. The intraclass correlation coefficients (ICCs) for PSS were 0.41 for the total sample, 0.45 for the control group, 0.38 for G1, and 0.39 for G2, indicating that the measure is sensitive to variability at both the between‐person and within‐person levels over time.

### 2.3 Data Analysis

The data were analysed in R (version 4.4.0) using RStudio (version 2023.06.2). Questionnaire total scores were considered continuous variables. Descriptives are provided using mean and standard deviation. Cronbach's α was computed to assess the internal consistency. The normality of the data distribution was assessed using both graphical methods (histogram and Q‐Q plot) and statistical tests (Shapiro‐Wilk, Pearson, and Anderson‐Darling). Data not showing normality were transformed (logarithmic or square‐root transformation), and normality was re‐checked. The equality of variances was tested using Bartlett's and Levene's tests. Between‐group differences (across generations) were assessed using appropriate tests based on the characteristics of the data (ANOVA, Kruskal‐Wallis test, or Welch ANOVA). When overall group differences were significant, *p*‐value adjusted post‐hoc analyses were performed: Tukey HSD method (for ANOVA), Benjamini‐Hochberg method (for the Kruskal‐Wallis test), and the Games‐Howell test (for Welch ANOVA).

Missing data on individual questionnaire items were imputed by the overall mean for the respective item, provided that the item had at least some non‐missing responses in the dataset (applied only four times in the dataset, all in PCL‐5). In cases when an entire questionnaire was missing for a participant, imputation was performed using the generation‐specific mean. Specifically, questionnaire data were missing for 5 subjects (2.8%) in the PCL‐5, 3 subjects (1.7%) in the SWLS, and 6 subjects (3.4%) in the Brief COPE questionnaires.

Modelling of the PSS total score over time was performed by marginal models using generalised least squares (GLS). This method is well‐suited for analysing longitudinal data with within‐subjects correlation structures, and we included an additional variance structure to account for differences in variability (heteroscedasticity) across groups. In these GLS models, the PSS total score was treated as a dependent variable, while day, generation, and baseline questionnaire outcomes were entered as independent variables. Gender was included as a covariate to account for potential differences in stress reporting. As age and other sociodemographic characteristics are integral to the definition of generational status, they were not included as covariates to retain model parsimony. Four different models were tested (see Results for details). The models were compared using the likelihood ratio tests, Cox & Snell *R* ^2^ coefficient, Akaike information criterion (AIC), and Bayesian information criterion (BIC) values.

## 3 Results

### 3.1 Between‐Group Differences in Demographics and the Baseline Assessment

The groups did not differ in gender composition (*p* = 0.229). Significant between‐group differences were found in age, relationship status, and monthly income (all *p* < 0.001). These differences were expected, as they reflect generational characteristics and demographic profiles of the study groups, with the control group not subdivided by generation.

Descriptive statistics for all the baseline measures are provided in Table 1, along with the results of between‐group comparisons. No significant differences in the baseline measures were found among the groups.

**TABLE 1:** Descriptive statistics for baseline measures and between‐group differences.

	Group						Between‐group differences
	Control		G1		G2		
	*M*	SD	*M*	SD	*M*	SD	
PCL‐5^ln^	0.97	0.75	1.01	0.65	1.08	0.70	F(2,159) = 0.415, *p* = 0.661, *η* ^2^ = 0.01
SWLS	24.88	5.65	23.08	6.22	22.18	5.71	χ^2^(2) = 5.807, *p* = 0.055, *η* ^2^ = 0.03
Brief COPE							
Problem‐focused coping	2.85	0.49	2.63	0.59	2.74	0.55	F(2, 158) = 2.566, *p =* 0.080, *η* ^2^ = 0.03
Emotion‐focused coping^sqrt^	2.50	0.35	2.39	0.44	2.43	0.49	F(2, 158) = 1.266, *p* = 0.285, *η* ^2^ = 0.02
Dysfunctional coping^ln^	1.85	0.37	1.86	0.48	1.96	0.56	F(2, 68.8) = 0.290, *p* = 0.749, ω^2^_adj_ = −0.01

Abbreviations: Control, Control group; G1, First generation; G2, Second generation; ln, logarithmic transformation; PCL‐5, PTSD Checklist for DSM‐5; sqrt, square‐root transformation; SWLS, Satisfaction with Life Scale.

### 3.2 Experience Sampling Method

There were 2414 observations (participants*days) for the PSS from 167 participants. A slight experimental fatigue was observed as the likelihood of completing the daily questionnaire decreased over time (*p* < 0.001). Furthermore, men were less likely to complete questionnaires compared to women. Table 2 provides the descriptive statistics of PSS total scores across the study period.

**TABLE 2:** Descriptives for PSS total scores across days and study group*s*.

	Total			Control			G1			G2		
Day	*n*	*M*	SD	*n*	*M*	SD	*n*	*M*	SD	*n*	*M*	SD
1	124	12.93	7.08	44	13.00	6.90	55	12.80	7.69	25	13.08	6.20
2	136	12.80	7.92	47	13.87	9.23	65	11.98	7.55	24	12.92	5.95
3	127	12.39	7.61	51	13.61	8.16	57	10.89	7.10	19	13.58	7.11
4	127	12.72	7.71	47	13.53	7.97	61	12.30	7.76	19	12.11	7.07
5	123	12.45	7.83	47	12.11	9.26	57	12.00	6.52	19	14.63	7.61
6	126	12.41	7.51	48	13.25	8.46	55	11.16	6.33	23	13.65	7.89
7	113	11.61	7.52	43	12.16	7.92	51	10.76	6.69	19	12.63	8.81
8	116	11.63	7.29	46	11.91	6.56	52	11.17	7.46	18	12.22	8.79
9	119	12.55	7.81	47	13.45	7.92	56	11.91	7.94	16	12.13	7.23
10	113	13.02	7.68	43	13.37	7.83	53	13.23	7.95	17	11.47	6.62
11	112	11.10	6.77	45	10.73	6.52	52	11.04	6.85	15	12.40	7.55
12	120	11.81	7.74	47	12.02	8.03	57	11.00	6.85	16	14.06	9.70
13	115	12.44	7.06	43	12.21	6.76	53	12.79	7.46	19	12.00	6.88
14	115	12.18	8.28	41	12.46	8.87	53	12.21	8.14	21	11.57	7.81
15	110	10.86	7.39	41	10.27	6.66	51	10.65	8.21	18	12.83	6.55
16	106	12.33	8.49	41	12.00	7.69	48	11.56	8.81	17	15.29	9.25
17	106	12.63	8.64	42	13.02	8.48	50	12.16	8.71	14	13.14	9.40
18	104	11.94	7.61	41	12.15	7.39	49	11.86	7.80	14	11.64	8.10
19	108	12.57	8.05	40	12.45	8.68	51	12.61	7.37	17	12.76	8.92
20	106	12.17	7.81	42	13.05	8.14	48	10.77	7.33	16	14.06	8.05
21	108	11.85	7.55	41	12.37	7.26	52	11.04	7.82	15	13.27	7.52

Abbreviations: G1, First generation; G2, Second generation.

Table 3 presents model fit indices for the following four marginal models: (1) Model 1: Differences in Mean PSS Total Scores Across Days; (2) Model 2: Differences in the Variability of PSS Total Scores Among Study Groups; (3) Model 3: Model 2 Extended With Baseline Questionnaire Scores; (4) Model 4: Reduced Model 3. Generally, the models showed good to acceptable fit, though Models 3 and 4 showed some deviations due to a few outliers.

**TABLE 3:** Model fit indices.

Model	LL	AIC	BIC	SE (df)	Standardised residuals (Range)	*R* ^2^
Model 1	−3364.40	6752.81	6822.25	1.207 (2414)	−2.94 to 2.63	0.346
Model 2	−3359.26	6746.52	6827.53	1.252 (2414)	−3.08 to 2.54	0.349
Model 3	−3319.27	6686.54	6825.33	1.139 (2414)	−3.50 to 3.02	0.370
Model 4	−3324.27	6686.54	6796.45	1.144 (2414)	−3.39 to 2.99	0.367

Abbreviations: AIC, Akaike information criterion; BIC, Bayesian information criterion; LL, Log‐likelihood; *R* ^2^, Cox & Snell *R* ^2^ coefficient; SE, Standard error of the estimate.

#### 3.2.1 Model 1: Differences in Mean PSS Total Scores Across Days

The first marginal model examined changes in mean PSS total scores across days, adjusted for gender and study group (PSS^sqrt^ ∼ Day + Gender + Group), to test for group differences in average daily stress perception. The model used an ARMA(5,1) correlation structure within random errors with maximum likelihood estimation.

**FIGURE 1:**
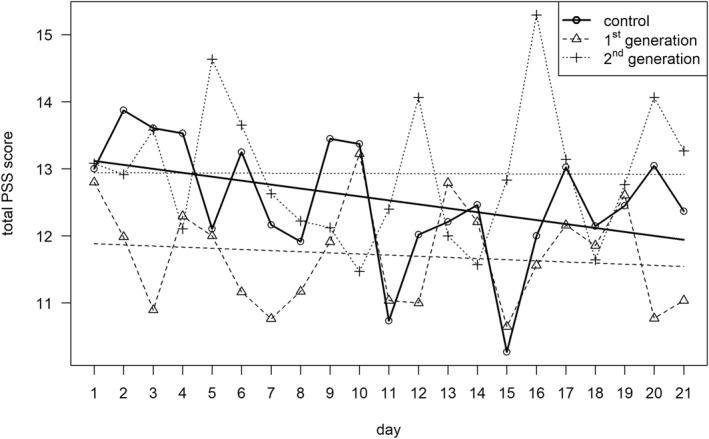
Mean PSS total scores over time by study group.

The analysis showed no significant differences in PSS^sqrt^ total scores between women and men (*b* = −0.228, SE = 0.133, *t* = −1.717, *p* = 0.086) or among the study groups (G1 vs. control: *b* = −0.210, SE = 0.145, *t* = 1.453, *p* = 0.146; G2 vs. control: *b* = 0.205, SE = 0.195, *t* = 1.051, *p* = 0.293; G2 vs. G1: *b* = 0.005, SE = 0.190, *t* = −0.026, *p* = 0.979). There was also no significant change in PSS total scores over time (*b* = −0.006, SE = 0.004, *t* = −1.73, *p* = 0.141). See Figure 1.

#### 3.2.2 Model 2: Differences in the Variability of PSS Total Scores Among Study Groups

Building on Model 1's focus on mean differences, Model 2 examined differences in the variance of PSS total scores across study groups by allowing for different variances between groups through a heterogeneous variance structure, while retaining the ARMA(5,1) correlation structure with maximum likelihood estimation.

Model comparison using a likelihood ratio test indicated that Model 2 provided a better fit than Model 1 (χ^2^(2) = 10.173, *p* = 0.006). Meaningful group differences were found. Compared to the control group, G1 showed approximately 8.26% lower variability in daily stress. G2 showed approximately 2.30% higher variability than the control group and 11.51% higher variability than G1. These results suggest group‐specific differences in the heterogeneity of stress responses, supporting the next step of examining whether baseline psychological factors contribute to these differences.

#### 3.2.3 Model 3: Model 2 Extended by Questionnaire Scores

Model 3 extended upon Model 2 by adding baseline questionnaire scores to assess their influence on daily PSS total scores. To account for a possible nonlinear relationship, both raw and square‐root‐transformed scores were included in the model (PSS^sqrt^ ∼ Day + Gender + Group + PCL‐5^sqrt^ + PCL‐5 + Problem‐focused coping^sqrt^ + Problem‐focused coping + Emotion‐focused coping^sqrt^ + Emotion‐focused coping + Dysfunctional coping^sqrt^ + Dysfunctional coping + SWLS^sqrt^ + SWLS). The model retained the heterogeneous variance structure from Model 2 and the ARMA(5,1) correlation structure within random errors with maximum likelihood estimation.

From all the baseline questionnaire scores, only PCL‐5 scores after square‐root transformation significantly predicted PSS^sqrt^ scores (*b* = 2.017, SE = 0.660, *t* = 3.056, *p* = 0.002), suggesting a nonlinear association between the severity of PTSD symptoms and daily stress (see Table 4).

**TABLE 4:** Regression coefficients for predictors of PSS^sqrt^ total scores in Model 3.

	*b*	SE	*t*	*p*
(Intercept)	−8.068	4.291	−1.880	0.060
Day	−0.007	0.004	−1.716	0.086
Gender				
Women	(ref.)			
Men	−0.032	0.117	−0.272	0.785
Generation				
Control	(ref.)			
G1	0.073	0.122	0.597	0.551
G2	0.060	0.178	0.399	0.734
PCL‐5^sqrt^	**2.017**	**0.660**	**3.056**	**0.002**
PCL‐5	−0.653	0.358	−1.825	0.068
SWLS^sqrt^	0.923	1.022	0.903	0.366
SWLS	−0.133	0.111	−1.200	0.230
Brief COPE				
Problem‐focused coping^sqrt^	−2.485	5.108	−0.486	0.627
Problem‐focused coping	0.543	1.598	0.340	0.734
Emotion‐focused coping^sqrt^	4.341	7.004	0.620	0.535
Emotion‐focused coping	−1.103	5.471	−0.484	0.628
Dysfunctional coping^sqrt^	10.239	5.603	1.871	0.061
Dysfunctional coping	−3.452	1.982	−1.741	0.082

*Note:* Bold indicates statistically significant results (*p* < 0.05).

Abbreviations: Control, Control group; G1, First generation; G2, Second generation; PCL‐5, PTSD Checklist for DSM‐5; sqrt, square‐root transformation; SWLS, Satisfaction with Life Scale.

Furthermore, the between‐group differences in variability of PSS total scores remained distinct after introducing baseline questionnaire scores into the model. Compared to the control group, G1 had 8.00% lower variability (vs. 8.26% in Model 2), and G2 had 3.95% higher variability (vs. 2.33% in Model 2). G2 also showed 12.98% (vs. 11.51% in Model 2) higher variability than G1. This indicates that being exposed to potentially traumatic events influences the range and spread of stress responses within these groups rather than mean stress levels.

#### 3.2.4 Model 4: Reduced Model 3 With Key Predictors

Model 4 streamlined Model 3 by retaining only the baseline questionnaires that showed relevance for predicting daily PSS score. Thus, the following scores were included: PSS^sqrt^ ∼ Day + Gender + Group + PCL‐5^sqrt^ + PCL‐5 + Dysfunctional coping^sqrt^ + Dysfunctional coping + SWLS^sqrt^. The model retained the heterogeneous variance structure from Model 2 and the ARMA(5,1) correlation structure within random errors with maximum likelihood estimation.

A likelihood ratio test comparing Models 3 and 4 showed no significant difference in model fit (χ^2^(5) = 7.13, *p* = 0.211), indicating that the reduction of predictors did not substantially reduce explanatory power. In contrast, comparing Models 2 (without baseline questionnaires) and 4 showed a significant difference (χ^2^(5) = 73.85, *p* < 0.001), indicating that PCL‐5, Brief COPE Dysfunctional coping, and SWLS are significantly associated with PSS daily total scores. Thus, the use of this more parsimonious final model was supported.

**FIGURE 2:**
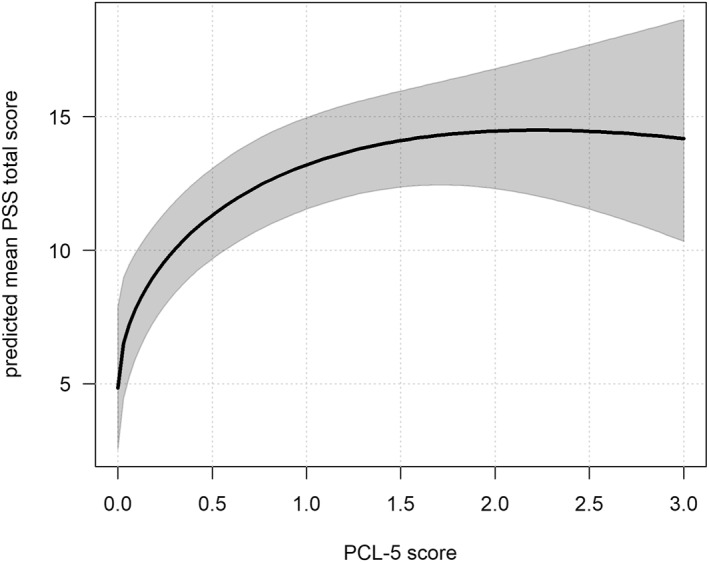
Association between predicted PSS total score and PCL‐5 score as estimated in Model 4. The thick line shows predicted mean values, and the grey band indicates 95% pointwise confidence intervals for the mean. The plots are illustrative, showing the overall patterns of the modeled relationships. Actual predicted values will vary across participants depending on their characteristics.

**FIGURE 3:**
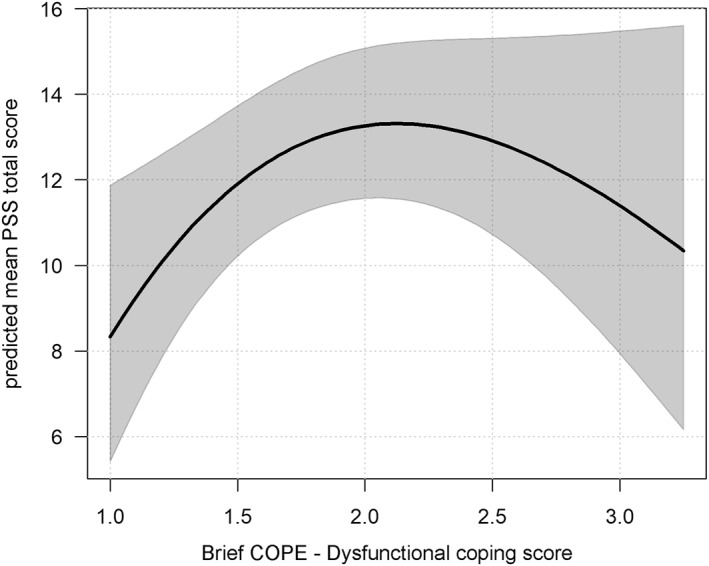
Association between predicted PSS total score and dysfunctional coping score as estimated in Model 4. The thick line shows predicted mean values, and the grey band indicates 95% pointwise confidence intervals for the mean. The plots are illustrative, showing the overall patterns of the modeled relationships. Actual predicted values will vary across participants depending on their characteristics.

**FIGURE 4:**
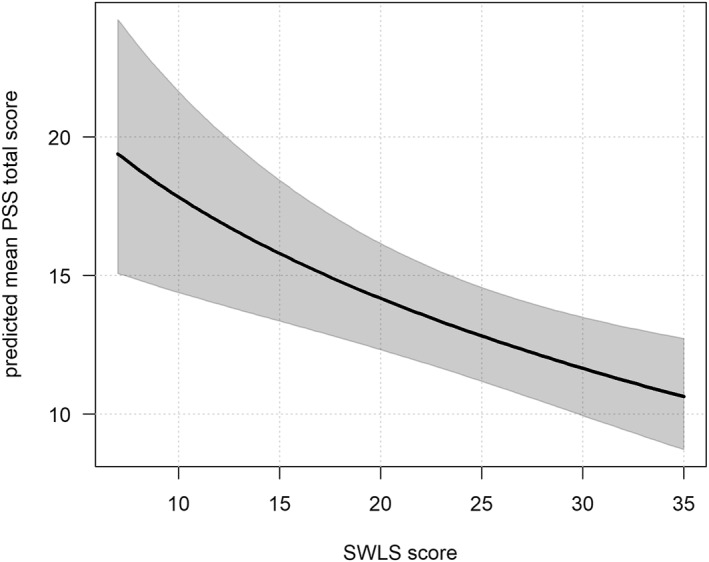
Association between predicted PSS total score and SWLS score as estimated in Model 4. The thick line shows predicted mean values, and the grey band indicates 95% pointwise confidence intervals for the mean. The plots are illustrative, showing the overall patterns of the modeled relationships. Actual predicted values will vary across participants depending on their characteristics.

The results of Model 4 are presented in Table 5. Higher PCL‐5^sqrt^ were associated with increased daily stress (*b* = 2.154, SE = 0.655, *t* = 3.290, *p* = 0.001). However, the raw PCL‐5 score showed a negative association (*b* = −0.743, SE = 0.355, *t* = −2.093, *p* = 0.036), suggesting a nonlinear relationship with a sharp increase in stress as the PTSD symptoms become more severe (see Figure 2). Similarly, Dysfunctional coping^sqrt^ showed a positive association (*b* = 11.995, SE = 4.611, *t* = 2.601, *p* = 0.009), while the raw score was negatively associated (*b* = −4.071, SE = 1.678, *t* = 2.426, *p* = 0.015) with PSS. As shown in Figure 3, the line follows an inverted U‐shaped relationship with stress levels peaking at moderate levels of dysfunctional coping. Higher SWLS^sqrt^ scores were linked to lower daily stress (*b* = −0.318, SE = 0.101, *t* = −3.149, *p* = 0.002). Figure 4 shows that stress decreases with greater life satisfaction.

**TABLE 5:** Regression coefficients for predictors of PSS^sqrt^ total scores in Model 4.

	*b*	SE	*t*	*p*
(Intercept)	−5.056	3.109	−1.626	0.104
Day	−0.007	0.004	−1.692	0.091
Gender				
Women	(ref.)			
Men	−0.035	0.113	−0.311	0.756
Generation				
Control	(ref.)			
G1	0.091	0.122	0.744	0.457
G2	0.069	0.177	0.391	0.695
PCL‐5^sqrt^	**2.154**	**0.655**	**3.290**	**0.001**
PCL‐5	**−0.743**	**0.355**	**−2.093**	**0.036**
SWLS^sqrt^	**−0.318**	**0.101**	**−3.149**	**0.002**
Brief COPE				
Dysfunctional coping^sqrt^	**11.995**	**4.611**	**2.601**	**0.009**
Dysfunctional coping	**−4.071**	**1.678**	**−2.426**	**0.015**

*Note:* Bold indicates statistically significant results (*p* < 0.05).

Abbreviations: Control, Control group; G1, First generation; G2, Second generation; PCL‐5, PTSD Checklist for DSM‐5; sqrt, square‐root transformation; SWLS, Satisfaction with Life Scale.

The variability across groups remained distinct in Model 4, matching the pattern observed in Model 3. Compared to the control group, G1 exhibited approximately 8.08% lower variability, whereas G2 showed approximately 3.43% higher variability, and 12.52% higher than G1.

## 4 Discussion

The present study examined daily stress variability among two generations of survivors of war in the former Yugoslavia and the non‐war‐exposed control group. The study focused on differences in baseline psychological measures (stress‐ and trauma‐related symptoms, coping, and life satisfaction), daily mean stress levels, and variability. We found no significant between‐group differences in the baseline measures. As expected, no significant differences in average daily stress levels were found. However, variability in daily stress levels showed distinct patterns across generations. Compared to the control group, G1 had lower stress variability, while G2 exhibited greater variability, with G2 showing the highest variability overall. Additionally, stress‐ and trauma‐related symptoms, dysfunctional coping, and life satisfaction predicted daily perceived stress. This suggests heightened sensitivity to daily stressors in G2, aligning with the latent vulnerability hypothesis (LVH; Shrira 2015; Shrira et al. 2024b, 2025). In contrast, lower variability in G1 may indicate stress tolerance (i.e., inoculating effects), but could also suggest reduced reactivity or culturally influenced suppression (Kelmendi and Hamby 2023; Palgi et al. 2015; Shrira et al., 2024b). These results suggest that the long‐term psychological consequences of war do not necessarily manifest in baseline or average levels of psychopathology but rather in altered reactivity and sensitivity to certain impulses, such as stress, even across generations.

As mentioned, previous studies found impaired mental health functioning, lower quality of life and satisfaction, but also adaptive coping capacities in survivors of conflict in the former Yugoslavia a decade after the war (Kelmendi and Hamby 2023; Matanov et al. 2013; Munjiza et al. 2017; Nickerson et al. 2014; Priebe et al. 2009, 2010; Thordardottir et al. 2020). The studies have also reported vulnerability to intergenerational phenomena and elevated stress‐ and trauma‐related symptoms in the second generation (Dikyurt 2023; Hyseni Duraku et al. 2023; Roch 2023). In contrast, we found no significant differences in baseline psychological measures between G1, G2, and the non‐war‐exposed control group. This may stem from methodological differences. Unlike previous studies conducted roughly a decade after the war, ours took place more than 20 years later. This gave the survivors more time for psychological adaptation, processing of their experiences, and potentially also for development of inoculating effects in G1, where prior exposure to potentially traumatic events may strengthen tolerance to everyday stress (Palgi et al. 2015; Shrira et al., 2024b). Moreover, previous studies typically lacked control groups and focused on prevalence rates or risk factors among survivors who remained in the Balkans or emigrated to non‐Slavic countries, facing additional migratory stressors, such as cultural and language barriers (Bogic et al. 2012; Dikyurt 2023; Hyseni Duraku et al. 2023; Matanov et al. 2013; Munjiza et al. 2017; Priebe et al. 2010, 2013; Roch 2023; Thordardottir et al. 2020).

Importantly, this study did not rely solely on baseline measures and cross‐sectional data; we also applied the experience sampling method to collect longitudinal data. While we found no significant differences in mean perceived daily stress levels over time among G1, G2, and the control group, variability in perceived stress differed, particularly when accounting for baseline stress‐ and trauma‐related symptoms.

G2 exhibited higher variability than both G1 and controls. Consistent with previous studies, this suggests that posttraumatic reactions, like stress sensitivity, may be intensified among the descendants, indicating transgenerational effects (Bowers and Yehuda 2016, 2020; Rosenheck and Fontana 1998; Maxwell 2014; Palgi et al. 2012; Shrira et al. 2024b, 2025; Shrira and Felsen 2021; Shrira and Felsen, 2021). Our results, thus, add to the growing evidence supporting LVH, which suggests that while G2 may not show elevated baseline stress, they are more stress‐sensitive and, therefore, more vulnerable to stressors. So far, most studies have focused on descendants of Holocaust survivors and their reactions to extreme potentially traumatic events, for example, the Hamas‐led attack on Israel on October 7, 2023 (Greenblatt‐Kimron et al. 2023; Shrira 2015; Shrira et al. 2024b, 2025; Shrira and Felsen 2021). Our study, however, demonstrated that this latent vulnerability to stress and alterations in stress regulation can be observed even in the context of the day‐to‐day life of G2, showing an increased sensitivity to stressors in general rather than only extreme or significant trauma.

In contrast to the daily stress sensitivity observed in G2, G1 exhibited lower variability in daily stress, suggesting more stable and habituated perceptions of everyday stress. Unlike Yom Kippur War veterans, who experienced reactivation or exacerbation of PTSD symptoms after exposure to a significant, reminding event (Shrira et al., 2024b), we did not observe such reactivity in the daily stress perceptions among G1. In our study, the lower variability in G1 aligns with findings that adults who have experienced potentially traumatic events may develop a reservoir of resources and strategies that stabilise stress responses or show overall dampening or rigidity in stress reactivity (Palgi et al. 2015; Shrira et al., 2024b).

Further insights come from the analysis of baseline psychological predictors of daily stress. Firstly, the severity of stress‐ and trauma‐related symptoms was non‐linearly associated with daily stress levels. Our results, aligned with the previous findings, emphasise the importance of considering the dimensions of symptom severity rather than focussing solely on the presence or absence of PTSD diagnosis per se (Zimmerman et al. 2018). Even subclinical levels of stress‐ and trauma‐related symptoms meaningfully shape daily perceptions of stress.

Secondly, the use of dysfunctional, avoidant, and non‐adaptive coping strategies was also significantly linked to perceived daily stress, following a nonlinear, inverted U‐shaped pattern. Specifically, the stress levels peaked at moderate levels of dysfunctional coping and slightly declined at the highest levels. This may indicate that those who rely on the use of dysfunctional coping engage in numbing or disengagement mechanisms that buffer the immediate perception of stress, though likely at other psychological costs. These findings are in line with prior research showing that avoidance, while potentially offering temporary relief, ultimately contributes to emotional dysregulation and poorer mental health outcomes (Ehlers and Clark 2000; Khailenko and Bacon 2024; Thompson et al. 2018).

Moreover, the cultural context may be considered here as well. Emotional suppression was regarded as a possibly positive coping strategy in the context of Southeast European cultures, associated with self‐regulation, endurance, and honour. However, it was also considered one of the elements of dysfunctional coping mechanisms (Anderson et al. 2019; Kelmendi and Hamby 2023). This ambiguity may help explain the nonlinear relationship observed in our study, as the boundary between adaptive and maladaptive coping may be fluid here. Relying on these culturally embedded mechanisms may initially heighten stress by limiting adaptive emotional processing, yet at the more extreme levels, it leads to disengagement from stress. These cultural dimensions may influence stress reactivity in everyday life and how people interact with their environment even decades after the war (Bronfenbrenner 1977; Harvey 2007). These factors may also contribute to explaining the generational differences observed in our study. G2, who was raised in changing cultural and migratory settings, was less influenced by the cultural norms of suppression as a valued strategy compared to G1. Consequently, G2 may show greater variability than G1 (Anderson et al. 2019; Kelmendi and Hamby 2023).

Lastly, we found higher life satisfaction associated with lower perceived daily stress. This supports previous studies that demonstrated life satisfaction's stress‐buffering, stress‐reducing, and stress‐containing effects (Gori et al. 2020; Ong et al. 2006; Thompson et al. 2018). It also shows the protective role of positive psychological resources in mitigating daily stress.

This study has both theoretical and practical implications. Theoretically, our findings expand the understanding of transgenerational trauma by revealing distinct patterns in daily stress perception across generations, i.e., heightened daily stress sensitivity in G2, aligning with the LVH and supporting it in populations beyond Holocaust survivors, and the lower stress variability in G1, consistent with more habitual reactions to stress in adults who delt with potentially traumatic events and potentially also the inoculation effect or cultural influence. Moreover, our results also show that the altered perceptions of stress are not limited to reactions to extreme stressors, as previously documented (e.g., Greenblatt‐Kimron et al. 2023; Shrira et al. 2024b, 2025), but are also reflected in everyday life through distinct patterns of variability in daily perceived stress. This highlights the need to consider how (transgenerational) psychological consequences of trauma manifest in ordinary, day‐to‐day life and the importance of considering the unique historical and socio‐cultural context of the war in the former Yugoslavia and subsequent ‘diaspora’ (Harvey 1996, 2007).

Practically, although individuals directly or indirectly affected by the war may not show elevated symptoms of psychopathology or distress decades later, these findings highlight the need for generation‐specific interventions. The higher levels of daily stress variability in G2 may reflect a latent vulnerability that puts them at increased risk for poorer mental health outcomes than their non‐war‐affected peers. This underscores the need for personalised interventions that go beyond symptom reduction and focus on enhancing stress regulation capabilities and promoting life satisfaction. In G1, where the stress perception was more stable, interventions might focus on supporting existing coping mechanisms, with culturally sensitive attention to suppression as a strategy that can be both adaptive and dysfunctional (Kelmendi and Hamby 2023). Moreover, multigenerational interventions with survivors of the war in the former Yugoslavia, sensitive to cultural nuances, may also be beneficial in reducing stress sensitivity when faced with adversities, even day‐to‐day life ones.

### 4.1 Strengths, Limitations, and Future Studies

This study has several strengths and limitations that should be noted. A significant strength is using ESM to capture daily perceived stress in a real‐life context over 21 days, reducing the recall bias. While the extended duration of the ESM data collection is a strength, it may also have contributed to slight experimental fatigue (Van Berkel et al. 2019; Wrzus and Neubauer 2023). To limit the daily burden on the respondents, we did not directly assess which daily stressors respondents encountered. Furthermore, relying on self‐reported measures may have introduced biases related to subjective perception, although this was mitigated by using intensive in‐the‐moment data collection.

Although the sample size was deemed adequate based on power analysis, some considerations should be mentioned. First, our power analysis was based on effect sizes from a study on Holocaust‐survivor families (Fňašková et al. 2021). Differences in historical, cultural, and migratory contexts may have influenced the magnitude and expression of the studied phenomena. Second, the natural attrition of participants in the ESM part of the study and the relatively small size of the G2 group meant that the sample size was at the lower bound of adequacy, which may have limited our ability to capture more subtle effects. Furthermore, the control group was not divided into generational cohorts as in some previous studies (e.g., Greenblatt‐Kimron et al. 2023, 2024; Shrira et al. 2025), and the G1 and G2 survivors were not drawn from the same families, limiting our ability to assess transgenerational effects directly.

While the models showed a good fit for the data, we could not validate them on an independent dataset; thus, there might be a possibility of overfitting. Future studies with larger and more diverse samples are needed to test the robustness of the models, ideally using a family‐based design to examine transmission effects directly. Longitudinal follow‐ups would help determine whether and how stress vulnerability and variability relate to future mental health outcomes. Moreover, incorporating physiological measures, such as cortisol levels or heart rate variability, could provide further evidence and complement the self‐reported data with objective markers of stress reactivity.

## 5 Conclusions

This study provides novel evidence that the lasting psychological consequences of the war in the former Yugoslavia may not be reflected in elevated daily stress levels or baseline psychopathology but instead are subtly expressed in altered perceptions and variability of daily stress even decades after the war. These effects were observed across generations. While the second generation born after the war showed increased variability, providing further support for the latent vulnerability hypothesis, the first generation directly exposed to war displayed lower variability. Stress‐ and trauma‐related symptoms, dysfunctional coping, and life satisfaction were key predictors of daily variability in perceived stress. This is the first study to assess these patterns in G1 and G2 survivors of the war in the former Yugoslavia. Taken together, these findings highlight the importance of providing personalised, targeted interventions that consider individual needs as well as the enduring legacies of broader socio‐cultural and family histories.

## Ethics Statement

Ethical approval for this study was obtained from the ethics committee of Masaryk University (approval code EKV‐2021‐076) on June 24, 2021.

## Conflicts of Interest

The authors declare no conflicts of interest.

## Data Availability

The data supporting the findings of this study are available upon reasonable request to the corresponding author.
